# Small airway dilation measured by endoscopic optical coherence tomography correlates with chronic lung allograft dysfunction

**DOI:** 10.1117/1.JBO.26.7.076005

**Published:** 2021-07-14

**Authors:** Jeanie Malone, Anthony M. D. Lee, Geoffrey Hohert, Roland G. Nador, Pierre Lane

**Affiliations:** a British Columbia Cancer Research Institute, Department of Integrative Oncology, Imaging Unit, Vancouver, BC, Canada; b University of British Columbia, Division of Respiratory Medicine, Faculty of Medicine, Vancouver, BC, Canada; c Vancouver General Hospital, Lung Transplant Program, Vancouver, BC, Canada

**Keywords:** optical coherence tomography, lung transplantation, chronic lung allograft dysfunction

## Abstract

**Significance**: Chronic lung allograft dysfunction (CLAD) is the leading cause of death in transplant patients who survive past the first year post-transplant. Current diagnosis is based on sustained decline in lung function; there is a need for tools that can identify CLAD onset.

**Aim:** Endoscopic optical coherence tomography (OCT) can visualize structural changes in the small airways, which are of interest in CLAD progression. We aim to identify OCT features in the small airways of lung allografts that correlate with CLAD status.

**Approach:** Imaging was conducted with an endoscopic rotary pullback OCT catheter during routine bronchoscopy procedures (n=54), collecting volumetric scans of three segmental airways per patient. Six features of interest were identified, and four blinded raters scored the dataset on the presence and intensity of each feature.

**Results:** Airway dilation (AD) was the only feature found to significantly (p<0.003) correlate with CLAD diagnosis (R=0.40 to 0.61). AD could also be fairly consistently scored between raters ($\kappa_{\text{inter-rater}}=0.48$, $\kappa_{\text{intra-rater}}=0.64$). There is a stronger relationship between AD and the combined obstructive and restrictive (BOS + RAS) phenotypes than the obstructive-only (BOS) phenotype for two raters (R=0.92,0.94).

**Conclusions:** OCT examination of small AD shows potential as a diagnostic indicator for CLAD and CLAD phenotype and merits further exploration.

## 1 Introduction

Chronic lung allograft dysfunction (CLAD) is the leading cause of death in lung transplant (LTx) patients who survive past the first-year post-transplant.^1^^,^^2^ CLAD affects nearly half of all patients 5-years post-LTx, reducing survival and quality of life and requiring increased monitoring and management.^3^ There are two primary phenotypes of CLAD: obstructive [bronchiolitis obliterans syndrome (BOS)] and restrictive [restrictive allograph syndrome (RAS)].^4^^,^^5^ LTx patients with CLAD can present with either phenotype or might evolve to a combined or mixed phenotype (BOS + RAS) over time.

The gold standard for definitive CLAD diagnosis is pulmonary function testing (PFT) that demonstrates a decline that is sustained for at least 3 months.^6^ The main drawback in using PFT to diagnose CLAD is the inherent delay in longitudinally testing lung function that is required to rule out acute conditions in LTx patients that cause transient fluctuations in pulmonary function, such as respiratory infections, acute rejection episodes (vascular or airway type of acute cellular rejection, organizing pneumonia pattern, diffuse alveolar damage, etc.), antibody-mediated rejection, main airway stenosis, or other extraparenchymal factors.^6^

Although the mechanisms of CLAD development are not fully understood, it is believed to progress from changes in the small airways and surrounding tissue.^6^ However, there is a lack of techniques that can provide relevant assessment of the small airways. The small airways are beyond the resolution of chest x-ray (CXR) and high-resolution computed tomography (HRCT). Transbronchial biopsies are often unreliable due to the heterogeneous nature of CLAD and challenges in obtaining biopsies containing small bronchioles.^7^ Thus, there is an unmet need for techniques that can directly and safely assess small airways for changes associated with CLAD.^3^

Using small catheters, endoscopic optical coherence tomography (OCT) is able to generate minimally invasive volumetric images of small airways without the use of ionizing radiation or contrast media.^8^ Endoscopic OCT has demonstrated potential in diagnosis in a number of lung disorders,^9^ such as lung cancers,^10^^–^^12^ chronic obstructive pulmonary disease,^13^^,^^14^ asthma,^15^^–^^17^ obstructive sleep apnea,^18^ and idiopathic pulmonary fibrosis.^19^ OCT can capture airway wall structure, remodeling, compliance, and alveolar structure.^20^ OCT extensions and multimodal imaging techniques can be used to examine vasculature^21^^,^^22^ and airway smooth muscle.^23^ OCT has also been precisely correlated to pulmonary histologic findings.^24^

Endoscopic OCT may be able to provide additional and possibly earlier optical biomarkers of CLAD. Its high resolution (10 to 15  μm, compared to ∼1 to 2 mm for HRCT) with a depth penetration of 1 to 2 mm allows for detailed imaging of bronchial and parenchymal structures. In addition, since it does not involve ionizing radiation, serial OCT imaging may allow for safely monitoring disease progression in CLAD over time.

## 2 Background

**Fig. 1:**
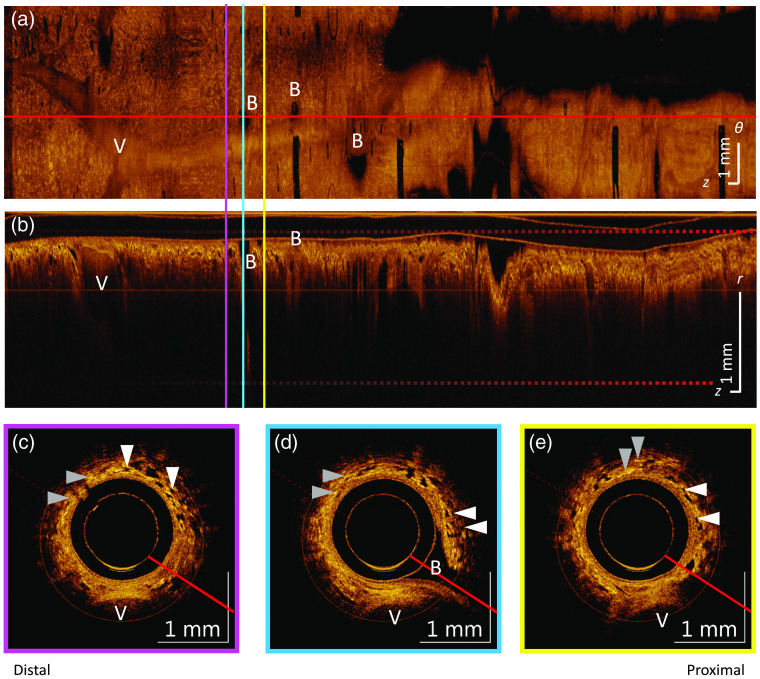
OCT of an airway from a non-CLAD LTx patient illustrative of normal-appearing airway structure. The airway images are oriented distal toward the left and proximal toward the right. (a) The *en face* mean intensity projection over the depth between the two dashed horizontal red lines shown in (b). (b) The longitudinal section taken along the red lines in the cross-sectional cuts and along the horizontal red line in (a). (c)–(e) Cross-sectional views from the corresponding pink, blue, and yellow vertically oriented slices in (a) and (b). Alveoli are demonstrated by small irregularly shaped dark regions (white arrows) and compressed alveoli by short bright lines (gray arrows). Airway branches (B) are comparable in size to the lumen from which they bifurcate. The lumen is tight around the sheath in the distal airway. There is a large blood vessel that runs parallel to the airway (V). Regularly spaced, vertically oriented dark bars in the *en face* projection are the black printed markers spaced every 10 mm on the catheter sheath. The θ scale bar in (a) represents dimensions projected onto the catheter circumference. The inner and outer surfaces of the outer sheath (1.5 mm OD) between appear as horizontally oriented lines at the top of the longitudinal sections, and round outlines in cross sections.

Figure 1 shows OCT of an airway from a non-CLAD LTx patient illustrative of normal-appearing airway structure acquired with a rotary-pullback catheter. The volumetric OCT images, collected in a cylindrical (r,θ,z) coordinate system with r, θ, and z coordinates corresponding to the A-line (i.e., depth into tissue), azimuthal, and airway axis directions respectively, are presented as an *en face* mean intensity projection (θ,z), a longitudinal section (r,z), and cross sections (r,θ).

In normal airway anatomy, the luminal area should steadily increase from distal to proximal. In the distal airway, the imaging catheter has expanded the airway such that it remains in contact with the catheter for some distance. In the mean intensity *en face* projection, regions where there is luminal contact with the imaging catheter appear bright whereas darker regions represent an air gap between imaging catheter and tissue. In Fig. 1, the dark region that spans ∼3.5  cm in the z-direction and ∼1  mm in the θ-direction indicates that, in this region, the airway lumen is larger than the imaging catheter.

Airway branches appear as round dark holes in the *en face* projection in the distal portion of the airway (label B in Fig. 1). These branches can be verified using longitudinal and cross sectional views to distinguish them from nonairway objects such as bubbles. A normal airway branch opening is comparable to the size of the lumen from which it bifurcates. For the 1.5-mm catheter used to acquire the images in Fig. 1, the longitudinal section shows the airway branch extends ∼1.5  mm in the z-dimension.

In the cross-sections and longitudinal sections, alveoli present as small (∼0.1  mm diameter) irregularly shaped polygonal regions devoid of scattering (dark). When not filled with air, alveoli can be visualized in the cross-section as short highly scattering (bright) line segments or crescents. Alveoli are present up to the luminal surface in the most distal part of the pullback where the airway is tight around the catheter sheath. In this situation, compression of the alveoli due to contact with the catheter is not unexpected. The presence of alveoli surrounding the distal airway appears as a lattice texture in the *en face* projection, whereas a blood vessel running parallel to the airway appears with smooth texture.

Mucus when present appears in small amounts and is translucent (lightly scattering). There also may be a few small duct-like structures (DLSs) that are dark and similar in appearance to alveoli in single cross-sections. Differentiation of these DLSs from alveoli comes from the fact that they are long features that extend parallel to the airway axis and thus show continuity across multiple successive cross-sections, whereas alveoli extend in the z-axis a length comparable to their azimuthal dimension.

## 3 Methods

### 3.1 Volunteer Recruitment

This study was approved by the Institutional Review Boards of the British Columbia Cancer Agency and the University of British Columbia (H14-00695). 54 volunteers were recruited from the Solid Organ Transplant Clinic from patients undergoing post-transplant bronchoscopy for routine surveillance (at 6 weeks, 6 months, and 1-year post-transplant) or in response to a sudden drop in lung function.

### 3.2 Imaging Protocol

The BC Cancer Research Institute’s Optical Imaging Lab has developed a catheterized OCT system with polarization diversity detection. The system has been described in detail previously.^25^ Briefly, the system uses a 50.4-kHz swept-source laser with a 20-mW polarized output centered at 1310 nm with a 100-nm bandwidth (SSOCT-1310, Axsun Technologies Inc., Billerica, Massachusetts). This is fed into a single mode 90% sample/10% reference Mach–Zehnder OCT interferometer.

In the sample arm, a fiber-optic rotary joint connects to a side-looking rotary-pullback imaging catheter (C7 Dragonfly imaging catheter, St. Jude Medical Inc., St. Paul, Minnesota). A custom rotary-pullback drive spins and retracts the imaging catheter to capture pullbacks up to 90 mm in length. During imaging, the catheter with an outer diameter of 0.9 mm is sheathed in closed-ended plastic tubing with an outside diameter of 1.5 mm, which allows for insertion in the instrument channel of commercial bronchoscopes. The polarization diversity detection was balanced as described previously,^25^ using the reference arm polarization controller and polarizer. The two polarization channels are collected with a fast digitizer (ATS9350, Alazartech, Pointe-Clare, QC, Canada) and processed with in-house data acquisition software.

Bronchoscopy was performed using standard procedure. After patient sedation, the bronchoscope was inserted into the trachea and navigated to a subsegmental airway (RB8, RB9, or RB10) in the right lower lobe. These subsegmental airways are routinely examined due to ease of access and ease of biopsy collection because the bronchoscope follows a relatively straight path.

The OCT catheter was advanced until fully in contact circumferentially with the airway wall then held stationary as the three-dimensional (3D) OCT volumetric pullback was acquired at a rotational speed of 100 Hz and a pullback speed of 4  mm/s for the maximum pullback length of 90 mm or until the imaging catheter was inside the bronchoscope. In cases where the imaging catheter did not return to the bronchoscope within the 90-mm pullback length, a secondary scan was taken after the catheter was retracted 20 to 50 mm to ensure overlap with the previous scan. In these instances, both pullback files together capture a complete image of the airway. This process was repeated to acquire images for the remaining subsegmental bronchi.

After the OCT pullbacks were acquired, the imaging catheter was removed and bronchoalveolar lavage (BAL) was performed for microbiological analysis. Transbronchial biopsies were collected in each subsegmental airway as per standard protocol but histopathologically processed together in the same cassette. Assessment of the combined biopsies was classified using the revised ABC scoring system for lung rejection.^26^

### 3.3 Feature Identification

By examining the CLAD airways in the OCT data set, rater 1, an expert in analysis of lung OCT images identified six features of interest that were notable when compared with a normal-appearing airway, namely: displacement of alveoli (DOA), emphysema-like alveolar enlargement (ELAE), airway dilation (AD), alveolar hyperinflation (AH), opaque mucous (OM), and DLSs.

**Fig. 2:**
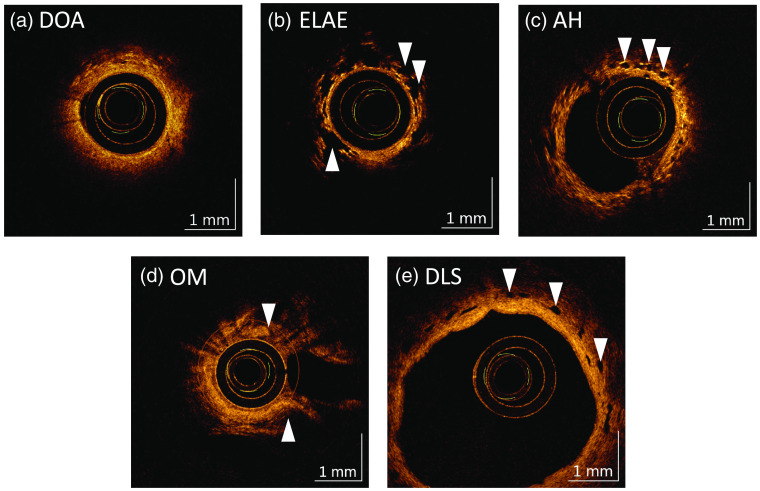
Cross-sectional views of features of interest; samples of each feature indicated by arrows. (a) DOA: increased spacing between alveoli with infill by uniform textured tissue. Some alveoli remain visible between 10 and 1 o’clock. (b) ELAE: large, irregular alveoli (7 to 2 o’clock). (c) AH: alveoli are spherical (11 to 2 o’clock and 3 to 5 o’clock). They are not compressed by catheter contact (10 to 5 o’clock). (d) OM: thick, opaque mucous obscures the tissue (11 to 2 o’clock and 4 to 6 o’clock). (e) DLS: round nonscattering structures (10 to 4 o’clock). Examination of adjacent cross-sections indicates they are extended structures that they run parallel to the airway.

Figure 2 shows cross-sectional views of five (DOA, ELAE, AH, OM, and DLS) of the six features of interest. Three of the features of interest describe alveolar changes present in the distal airway: DOA [Fig. 2(a)], ELAE [Fig. 2(b)], and AH [Fig. 2(c)]. The presence of some of these features may preclude the assessment of the others, although they may all be present in different portions of the airway. DOA is characterized by a lack of alveoli in a region where alveolar texture should be present. In this feature, there is increased spacing between the alveoli and infill by uniform, lightly scattering material. The density of visualized alveoli decreases and in extreme cases, normal-appearing alveoli appear to be displaced entirely. It was hypothesized that this feature could be atelectasis, luminal thickening, or fibrosis displacing alveolar structures. The ELAE feature is characterized by remarkably enlarged alveoli, resembling those we have seen in patients with mild to moderate emphysema. It was suspected that this feature could represent diffuse alveolar damage. For the AH feature, rather than the anticipated polygonal or crescent-like structures, individual alveoli appear as spherical voids that do not compress even in response to contact pressure from the catheter. This feature was thought to represent air-trapping, an indicator of CLAD, which is visualized by mosaic attenuation in HRCT.

Although some mucous may be present in normal airways, it generally is translucent (sparsely scattering) and is only present sporadically. The OM feature [Fig. 2(d)] is characterized by copious amounts of thick, OM that obscures underlying tissue and occludes airway branches. In the *en face* projection OM appears as highly scattering material that is frothy with large air bubbles and obscures normal luminal surface topology. This feature was suspected to be related to inflammation and/or fibrotic response and was explored for potential in distinguishing acute infection.

In normal appearing airway, DLSs are similar in size to alveoli (∼0.1  mm) with no more that 1 or 2 per airway. The DLS feature [Fig. 2(e)] is characterized by the excessive presence (>5 per airway) and/or larger size of DLSs (>0.3  mm). This feature was suspected to be related to the mucous feature.

**Fig. 3:**
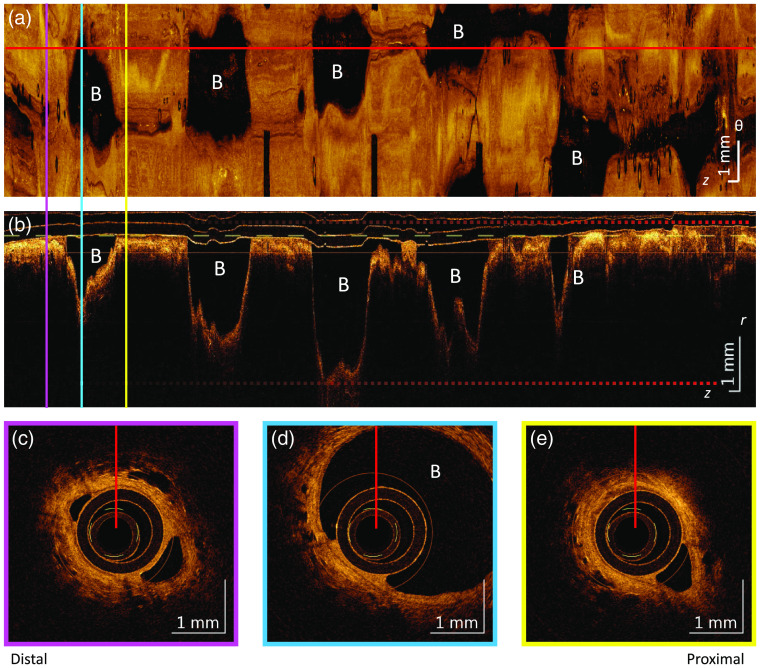
Airway demonstrating AD feature. (a) The *en face* mean intensity projection over the depth between the two dashed red lines shown in (b). (b) The longitudinal section taken along the red lines in the cross-sectional cuts and along the horizontal red line in (a). (c), (d), and (e) Cross-sectional views from the corresponding pink, blue, and yellow vertically oriented slices in (a) and (b). All airway branches (B) display AD.

Figure 3 shows an airway that displays the AD feature, hypothesized to visualize bronchiectasis or air-trapping. In this example, AD can be ascertained by examining the cross-section images distal [Fig. 3(c)] and proximal [Fig. 3(e)] to an airway branch [Fig. 3(d)]. Away from the airway branch, the luminal area is a little larger than the catheter cross-sectional area. However, at the airway branch, the luminal area expands much greater than twice the luminal area from which it originates. As seen in the longitudinal section, the dilation of the airway lumen near the airway branch extends for almost 5 mm along the z dimension, much larger than the diameter of the airway from which it originates. In the *en face* projection, AD appears as very large dark regions around each of the airway branches. In this example, AD is associated with an airway branch, whereas in other examples within the data set, AD occurs where there is no visible airway branch.

### 3.4 Feature Scoring

Rubrics were developed to assist assessing the dataset for the presence and severity of the identified features. DOA, ELAE, and AD were assessed on a scale of 0 to 2 as described in Table 1. Features that could only be assessed on presence or lack thereof (AH, OM, DLS) were assessed on a binary scale as described in Table 2.

**Table 1:** Scoring rubric for graded OCT features of interest.

Feature	Scored 0 (none)	Scored 1	Scored 2
DOA	Normal alveoli spacing: alveoli are densely packed in the airway wall. Alveoli can be compressed by the catheter, appearing as bright, short lines in the airway wall.	Alveoli partially displaced by uniformly scattering material.	Alveoli fully displaced by uniformly scattering material.
ELAE	Normal alveoli size: <200 μm in diameter. Alveoli can be compressed by the catheter, appearing as bright, short lines in the airway wall.	Enlarged, irregularly shaped alveoli along some (<50%) of the airway.	Enlarged, irregularly shaped alveoli present along most (>50%) of the airway.
AD	Normal airway appearance: steadily increasing luminal size from distal to proximal airway. Luminal enlargement around an airway branch is similar in size to the airway from which it bifurcates.	Moderate airway enlargement of distal relative to proximal airway or some (<50%) airway branches have luminal enlargement over lengths that are larger in size than the airways from which they bifurcate.	Marked airway enlargement of distal relative to proximal airway or many or all (>50%) airway branches have luminal enlargement over lengths that are larger in size than the airways from which they bifurcate.

**Table 2:** Scoring rubric for binary OCT features of interest.

Feature	Scored 0	Scored 1
AH	Normal alveoli shape: inflated alveoli appear polygonal. Alveoli that are uninflated or compressed by the catheter appear as bright, short lines in the airway wall.	Entirely spherical alveoli; maintain roundness irrespective of compression from the catheter.
OM	Normal mucous appearance: low scattering and not occluding airway.	Copious OM occluding the airway and/or branches.
DLSs	None to 1 to 2 per airway DLSs observed running parallel to the airway.	Numerous >3 per airway or large (>0.3 mm) DLSs observed running parallel to the airway.

Four raters were recruited who had no previous experience in CLAD diagnosis. Three of these raters had minimal experience in the assessment of OCT. The rater (AL) with significant OCT experience prepared the training materials that were used by all raters during the assessment of the dataset. This training including scoring rubrics (Tables 1 and 2), an overview of endoscopic OCT interpretation, annotated example projections of each feature, and the associated video pullbacks for each example.

To capture the volumetric nature of the OCT, two video pullbacks were provided per sample: one that serially presented cross-sections correlated with z position on the *en face* projection, and one that serially presented longitudinal sections correlated with θ position on the *en face* projection. In cases where multiple pullbacks were needed to capture the full length of one airway, all video pullbacks for each airway were provided and raters were asked to assess them together to produce a single overall airway score.

Raters were provided an anonymized, randomized set of videos for each airway and asked to score each for the presence of the six features in the rubrics. Raters scored the dataset independently and in a randomized order, blinded to clinical status. One rater repeated scoring of the feature most strongly correlated with CLAD three times over a period of several weeks to allow for assessment of intrarater consistency.

### 3.5 Statistical Analysis

Each rater produced score vectors of the six features for each airway of each patient. A cumulative patient score vector [ΣDOA, ΣELAE, ΣAD, ΣAH, ΣOM, ΣDLS] was generated by summing the individual airway scores for each feature. This cumulative score vector was then used for subsequent analysis against clinical status. Each rater was assessed separately.

The correlation between features and clinical status was assessed using the Pearson correlation coefficient (R). As the assessment of clinical status (CLAD status, CLAD phenotype, infection status, and histopathologic findings) is conducted for the LTx rather than for each individual airway, features were assessed using cumulative score vectors rather than individual airway scores. For analysis of features of interest against histopathology, any ungradable or not collected biopsy samples were removed from the dataset before calculating correlations to prevent finding relationships with nonresponse. The area under the curve (AUC) was calculated for each receiver operator characteristic (ROC) curve to examine the diagnostic potential of significantly correlated features of interest.

Prevalence of the features of interest was calculated as a percentage of score (0,1,2) out of the total dataset, using per-airway scores. Similarly, Fleiss’ Kappa (κ) was calculated using per-airway scores to examine interrater and intrarater reliability.

Potential confounding factors were analyzed using two-tailed Mann–Whitney U test (continuous variables) and two-tailed Fisher’s exact test (all others). This included gender, age, time from transplant, clinical infection status, and histological findings.

A significance value of p<0.05 was selected for all analyses. In analyses derived from multiple raters, the Bonferroni correction for multiple comparisons was applied such that $p_{\text{Bonferroni}}<0.007$.

## 4 Results

### 4.1 Participants and Descriptive Data

54 patients were imaged between 2016 and 2018. Demographic information, infection status, and histopathology results are described in Table 3. CLAD status and phenotype at time of imaging were assessed by a pulmonologist specializing in lung transplant [RN] using the 2014 international clinical guidelines (ISHLT/ATS/ERS).^27^ Participants who met these diagnostic criteria, including demonstrating a sustained decline in PFT measurements not attributed to another cause, were included in the CLAD group. CT studies were conducted as in normal clinical practice to assess phenotype. Participants who did not meet the diagnostic criteria for CLAD were placed in the non-CLAD group.

**Table 3:** Summary statistics. Significant values (p<0.05) are bolded.

		All cases (n=54)	Non-CLAD (n=45)	CLAD (n=9)	p-value
Gender					1.000
	Male	33 (61.1%)	27 (60%)	6 (66.6%)	
	Female	21 (38.9%)	18 (40%)	3 (33.3%)	
Mean time since transplant ± SD (months) [range]		32.8±73.3 [1 to 454]	12.7±16.1 [1 to 90]	133±143 [21 to 454]	<0.001
Mean age ± SD (years) [range]		60.4±16.8 [22 to 8]	58.4±15.3 [22 to 72]	69.9±21.5 [36 to 84]	0.159
Infection status					0.259
	Clinically relevant infection	6 (11.1%)	4 (8.9%)	2 (22.2%)	
	No clinically relevant infection	48 (88.9%)	41 (91.1%)	7 (77.8%)	
Histology A: acute rejection					1.000
	A0 (none)	39 (72.2%)	34 (75.6%)	5 (55.6%)	
	A1 (minimal)	5 (9.3%)	4 (8.9%)	1 (11.1%)	
	A2 (mild)	2 (3.7%)	2 (4.4%)	0 (0%)	
	A3 (moderate)	0 (0%)	0 (0%)	0 (0%)	
	A4 (severe)	0 (0%)	0 (0%)	0 (0%)	
	AX (ungradable)	2 (3.7%)	1 (2.2%)	1 (11.1%)	
	ND (no sample)	6 (11.1%)	4 (9.0%)	2 (22.2%)	
Histology B: airway inflammation					0.105
	B0 (none)	37 (68.5%)	34 (75.6%)	3 (33.3%)	
	B1R (low grade)	1 (1.9%)	0 (0%)	1 (11.1%)	
	B2R (high grade)	0 (0%)	0 (0%)	0 (0%)	
	BX (ungradable)	10 (18.5%)	7 (15.6%)	3 (33.3%)	
	ND (no sample)	6 (11.1%)	4 (8.9%)	2 (22.2%)	
Histology C: bronchiolitis obliterans					1.000
	C0 (absent)	37 (68.5%)	33 (73.3%)	4 (44.4%)	
	C1 (present)	0 (0%)	0 (0%)	0 (0%)	
	CX (ungradable)	11 (33.3%)	8 (17.8%)	3 (33.3%)	
	ND (no sample)	6 (11.1%)	4 (9.0%)	2 (22.2%)	

Of the CLAD cases, six cases were BOS phenotype and three cases were the mixed BOS + RAS phenotype. BAL results are representative of infection status, which is inclusive of fungal colonization as well as viral and bacterial infections. BAL results were classified by the clinician (RN) as clinically relevant or the result of contamination from oral cavity flora. Histology was classified using the revised ABC scoring system for lung rejection.^26^

Time from transplant has a significant association with CLAD status as expected: the sample set was comprised of either <1 year surveillance patients who cannot be assessed for CLAD until a PFT baseline has been established, or >1 year adverse PFT event patients. In addition, CLAD incidence increases with time from transplant.^1^

### 4.2 Airway Dilation as a Predictor of CLAD Status

CLAD status was examined using Pearson’s correlations against the cumulative feature scores for each patient. AD was the only feature that was found to significantly and positively correlate with CLAD, with relatively weak R-values from 0.40 to 0.61, as demonstrated in Table 4. None of the other features correlated significantly with CLAD, with several features including ELAE and AH demonstrating nearly no relation (R∼0). Nonsignificant findings can be found in Table S1 in the Supplemental Material.

**Table 4:** Significant Pearson correlations (R) between CLAD status and cumulative feature score for each rater. Prevalence refers to the percentage of the dataset (n=54) for each corresponding score (0,1,2), calculated per-airway. Kappas represent the overall rater reliability. Correlations that meet the significance criteria after Bonferroni correction ($p_{\text{Bonferroni}}<0.007$) are bolded.

Feature	R (p-value)				Mean prevalence of scores ± SD	$\kappa_{\text{overall}}$ 95% CI
	Rater 1	Rater 2	Rater 3	Rater 4		
AD	**0.59** (<0.001)	**0.40** (0.003)	**0.49** (<0.001)	**0.61** (<0.001)	0:74%±2	**0.48** (<0.001); 0.47 to 0.50
					1:21%±3	
					2:5%±2	

Most features were present in <25% of the dataset, as described by the mean prevalence column in Table 4 and Table S1 in the Supplemental Material. The standard deviation of the mean prevalence provides an indication of consistency between raters. As expected, even with uniform training there is some variation in assessment for each rater. Of the graduated features, AD has the highest consistency.

Fleiss’ kappa values for all features are significant and have relatively tight 95% confidence intervals. The kappa values indicate AD can be moderately consistently rated, and it is more consistently assessed than most other features of interest. DOA and OM are fairly consistently recognized with a κ of 0.39. ELAE demonstrated the worst consistency with a κ of 0.26 among the four raters, due to a discrepancy in prevalence between rater 3 and the other raters, which is captured in the wide standard deviations of the mean prevalence.

Intrarater correlations for threefold assessment of AD separated by several weeks are shown in Table 5. The correlations are only slightly stronger than the interrater trial, and despite greater agreement still demonstrate low R-values from 0.50 to 0.61. Scores of 1 (low AD) and 2 (high AD) are slightly more prevalent than the interrater mean scores and have a very tight standard deviation. The kappa value indicates improved agreement: κ=0.64 is indicative of substantial agreement rather than moderate agreement. Overall, the AD feature can be consistently scored by the same rater over time.

**Table 5:** Pearson correlations (R) between CLAD status and cumulative AD (ΣAD) score per lung for intrarater score sets. Prevalence refers to the percentage of the dataset (n=54) for each corresponding score (0,1,2), calculated per-airway. Correlations that meet the significance criteria after Bonferroni correction ($p_{\text{Bonferroni}}<0.007$) are bolded.

Feature	R (p-value)			Mean prevalence of score ± SD	$\kappa_{\text{overall}}$ 95% CI
	Score set 1	Score set 2	Score set 3		
ΣAD versus CLAD	**0.61** (<0.001)	**0.50** (<0.001)	**0.51** (<0.001)	0:77%±0.5	**0.64** (<0.001) 0.62 to 0.66
				1:16%±0.5	
				2:7%±0.7	

### 4.3 Airway Dilation as a Predictor of CLAD Phenotype

**Fig. 4:**
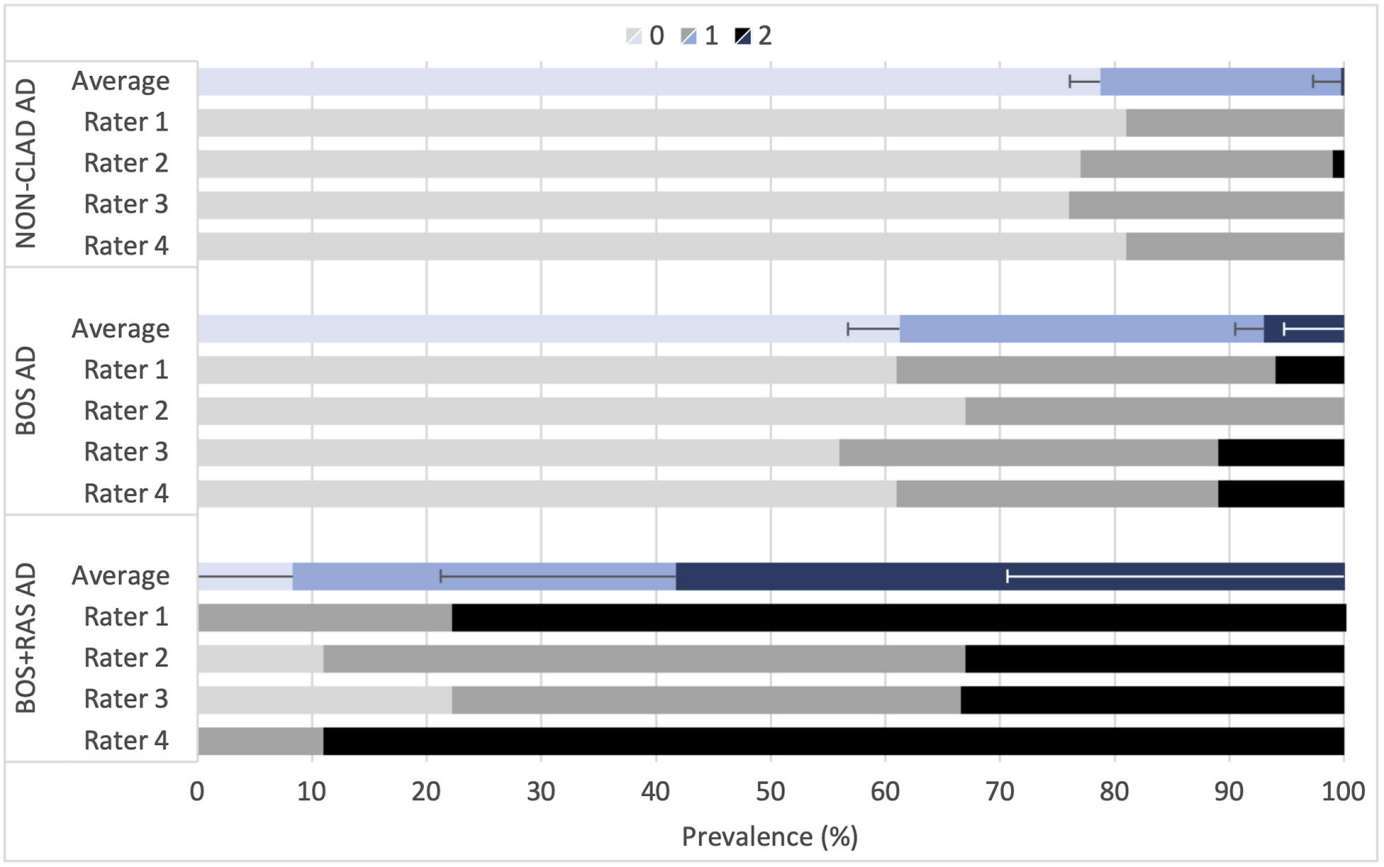
Prevalence of scores per airway for the AD feature, separated into non-CLAD (n=135), BOS-only (n=18), and BOS + RAS (n=9) phenotypes.

An assessment of the AD score prevalence in non-CLAD cases and CLAD phenotype is shown in Fig. 4. The combined BOS + RAS phenotype has higher AD scores than the BOS-only phenotype. Approximately 20% of the non-CLAD airways were scored 1, with only one rater identifying one airway (0.7%) as highly dilated (score 2). In contrast, 40% of the BOS-only airways demonstrated a nonzero AD score with 6% scoring highly dilated. In the BOS + RAS cases, over 90% of airways have a non-zero AD score with 58% of airways scoring highly dilated. As before, no significant relationship was found with any of the other features of interest.

Analysis of the AD feature against the CLAD phenotypes shows a significant positive correlation with the BOS + RAS phenotype for rater 1 and rater 4 (R=0.94, 0.92). This is a much stronger correlation than AD scoring distinguishing between CLAD and non-CLAD (R=0.40 to 0.61); however, this correlation is not significant for all raters.

### 4.4 Correlations with Other Clinical Findings

There are occasional significant correlations between the features of interest and other clinical findings including infection and histopathology status; however, none met the significance threshold for more than one rater.

### 4.5 Receiver Operating Characteristic Curve

**Fig. 5:**
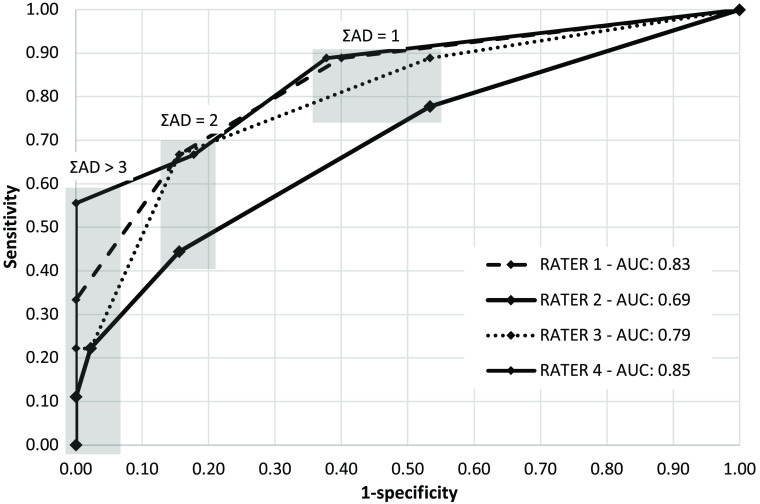
ROC curve for AD feature. ΣAD=1, 2, 3 indicates increasing diagnostic threshold scores for the cumulative AD score.

The ROC curves displaying sensitivity and specificity for each rater’s cumulative AD score is shown in Fig. 5. The AUC ranges from 0.69 to 0.85 between the raters. In comparison, the use of HRCT findings such as mosaic perfusion, bronchiectasis, and bronchial wall thickening to diagnose BOS without PFT findings have sensitivities of 4% to 25%.^28^ While PFT assessment of CLAD is highly sensitive to longitudinal lung function drops, this requires demonstration of a consistently lowered function over time and does not provide early indication of CLAD. Transbronchial biopsies have high specificity but very low sensitivity and very low diagnostic yield for CLAD. Features identified in CT, CXR, and HRCT provide limited accuracy unless examining well-established CLAD cases^6^^,^^27^ in combination with PFT findings.

## 5 Discussion

We present a pilot study exploring the potential of using endoscopic OCT to aid CLAD diagnosis. Six abnormal OCT features of interest were identified. Scoring rubrics and training materials were developed, and four raters independently assessed each feature in a dataset of 54 LTx patients. Analyses of the rater score sets provided four findings: first, that there is a weakly positive correlation between AD and CLAD, and that this feature can be more consistently rated than other features of interest, with moderate inter-rater agreement and substantial intrarater agreement. Second, there is a positive correlation between AD and the BOS + RAS phenotype. Third, there are no significant correlations among DOA, ELAE, AH, OM, or ED, and CLAD. Fourth, there is no repeatable relationship between histopathology data and any of the features of interest. These findings are limited by the size and composition of the dataset as discussed below.

### 5.1 Weak Correlation Between AD and CLAD

The weak correlation between AD and CLAD fits some of the characteristics of CLAD progression seen in radiography. The AD feature itself encompasses several changes: luminal expansion of the small airway being imaged and abnormally large airway branch openings. AD likely reflects radiographically observed bronchial dilation or bronchiectasis. Previous studies have found bronchial dilation and bronchiectasis in some BOS cases,^29^ with CT findings demonstrating a weak correlation between BOS stage and degree of bronchial dilation,^30^ in general thought to be associated with more advanced cases of obstructive CLAD. More prominent and earlier radiographic features of BOS include mosaic attenuation in HRCT^27^ related to air trapping accentuated on expiratory images, which could also relate to the airway wall damage leading to focal dilation of small airways with early collapsibility upon expiration.

Although the AD feature is the most consistently rated feature explored in this work and the associated Kappa values demonstrate some improvement in consistency in the intrarater assessment, there remains a relatively weak correlation between AD and CLAD. The lack of substantial improvement in the intrarater trial indicates that the strength of the correlation between this feature and CLAD is not simply improved by increasing rater reliability. Further refinement of the qualitative characteristics assessed by the AD feature in this work may provide more utility.

### 5.2 Stronger Correlation Between AD and BOS + RAS Phenotype

AD demonstrates a much stronger relationship with the BOS + RAS phenotype than the BOS-only phenotype, although this finding is substantially limited by the sample size of CLAD-positive cases. This relationship is unexpected at first glance, as one would expect the BOS phenotype to be more associated with airway changes such as dilation and bronchiectasis. BOS is characterized by changes ranging from excess subepithelial tissue, loss of smooth muscle, fibrotic luminal obliteration, inflammatory responses, and autoimmune reactions^6^^,^^27^ of the bronchioles. RAS tends to affect the interstitium but can extend to the terminal bronchioles through diffuse alveolar damage or organizing pneumonia pattern in the earlier stages, leading to interstitial fibrosis reminiscent to the histological diagnosis of pleuroparenchymal fibroelastosis.^29^^,^^31^ Persistent and progressive radiographic opacities on HRCT or CXR are reflective of these parenchymal changes, nonresolving ground glass opacities, reticulation, dense fibrosis/scarring, pleural thickening are requirements for RAS diagnosis.^6^ It is possible that this increased AD is due to traction bronchiectasis, which has been noted in some RAS cases.^29^ Furthermore, bronchiolitis obliterans histologic changes have been frequently observed adjacent to the fibrotic changes in explant lungs from patients with RAS.

It is noted that the sample set in this study only BOS and mixed (BOS + RAS) phenotype cases, and additional examination of predominantly RAS phenotype cases is needed to better understand the relationship between AD and phenotype. Due to the sample size of BOS + RAS cases in this dataset only two raters had a statistically significant relationship, and rater 2 does not appear to follow prevalence trends of the other raters, which could represent a failure in training. Further study with a larger cohort is necessary before conclusions can be drawn but points to AD in CLAD phenotype as a potential area to explore in future work. An examination of PFT data alongside OCT would allow for exploration of the underlying mechanism behind this relation.

### 5.3 No Correlation Between CLAD and Other Features of Interest

It is surprising that none of the other features of interest correlated with CLAD cases, as they were expected to capture common characteristics of CLAD. DOA could have been related to luminal thickening or fibrosis. ELAE or AH could have been related to diffuse alveolar damage or the air trapping as seen in HRCT. Positive results may have been washed out of the dataset through the cumulative score vector, but even analysis of an airway-by-airway correlation does not provide any relation other than AD. The heterogeneity of CLAD presentation may also have obscured results if the specific airways imaged were not affected. LTx patients, particularly the <1 year post-transplant cases, which are a prominent part of our non-CLAD samples, are undergoing many changes including airway remodeling, inflammation responses, and immune response, which may have caused the presence of these features within the non-CLAD cases. Studies with larger cohorts would allow for a more conclusive assessment of these features, from this limited pilot work they do not demonstrate promise.

### 5.4 No Correlations Between Infection or Histopathology Data and Other Features of Interest

The lack of consistent relation between features of interest and histopathology results is exemplary of the challenges of the biopsy ABC scoring system: there are very few positive histology results present in this dataset and may be many false negatives. Although we may expect a relationship between the histology C (bronchiolitis obliterans, BO) and the BOS phenotype,^26^ histopathologic evidence of BO is uncommon due to the heterogeneity of progression and limitations of transbronchial biopsy.^6^ There remains a need for further exploration of cases that demonstrate pathologic findings to understand whether they can be visualized with OCT, and whether OCT can distinguish between pathologic findings and CLAD.

### 5.5 Clinical Relevance

In this clinical application, endoscopic OCT fits within routine bronchoscopies and does not require radiation or contrast media, allowing for the potential of repeat study of specific distal airways. It is more invasive than CXR or HRCT and does not allow assessment of the entire lung, which limits utility due to the heterogeneous nature of CLAD, but the resolution of OCT allows for the examination of smaller changes in the small airways and surrounding parenchyma that may correlate with earlier diagnosis. It is conceivable that OCT would be able to detect small airway changes earlier in the disease process warranting for close monitoring and possible treatment interventions for CLAD. It is unlikely that endoscopic OCT would be conducted outside the circumstance of routine bronchoscopies or replace PFT in CLAD diagnosis, but it may be used as an adjunct to the current diagnostic protocols.

### 5.6 Limitations and Future Directions

Overall, the OCT examination of AD demonstrates some potential relation to CLAD status and merits further investigation. None of the other features of interest presented such potential. While the ROC shown in Fig. 5 demonstrates promise, this feature may be further improved through quantitative approaches. Separately quantifying luminal expansion and airway branch enlargement would allow for a deeper examination of the interplay between these two features and greater understanding in how they relate to CLAD progression and phenotype. Other airway features that may relate to the rater assessment of AD such as diameter, perimeter, or regularity airway branches could also be explored. An automated quantitative assessment would remove concerns about rater reliability, though the preliminary findings in the intrarater study indicate that improved reliability is not sufficient to improve correlation between the AD feature as described in this work to CLAD.

This work represents a pilot study and as such the findings are substantially limited by the available dataset: the low number of CLAD-positive participants especially given the heterogeneous nature of CLAD, the low incidence of positive histological findings, and the lack of matched PFT and radiology to each case. Further study with a larger cohort is required before conclusions can be drawn about the diagnostic potential of OCT in this application, but this work has indicated some potential areas for exploration. A longitudinal study comparing OCT features to changes in PFT would allow for understanding of whether serial imaging with endobronchial OCT can capture CLAD progression. Further study of cases of predominantly RAS phenotype would allow for a better understanding of the potential of OCT to be used in phenotypic distinction. A cohort that demonstrates a broad range of pulmonary conditions, including cases with positive pathologic findings, is required to assess the potential of OCT in distinguishing CLAD from confounding conditions.

## 6 Conclusions

We present a pilot study using endoscopic OCT to perform 3D imaging of the small airways in lung transplant patients. Our findings show that there are distinctive, consistently recognizable OCT features, which can be identified by those without previous clinical experience in lung diagnostics. These features included alveolar changes such as DOA, hyperinflation, or emphysema-like enlargement as well as OM, and excessive DLSs.

One of the six features of interest, only AD, characterized by luminal expansion and enlargement around airway branches, was significantly but weakly correlated with CLAD status. This feature demonstrated moderate interrater agreement and substantial intrarater agreement. AD has a stronger correlation with the mixed obstructive and restrictive phenotype (BOS + RAS) when compared with the singular obstructive phenotype (BOS), but this finding is limited by the small sample size of CLAD-positive cases. No relation was found between CLAD status and the other five features of interest. Examination of infection status and histological data found no consistent and significant relation with any of the features.

The conclusions that can be drawn from this pilot study are limited but support further exploration of OCT in this application. Areas for future work include quantification approaches for features related to small AD, an examination of OCT against PFT and/or radiographic data, longitudinal studies to assess utility for early detection, and larger cohort studies to better understand diagnostic potential given the heterogeneity of CLAD and other potentially confounding pulmonary conditions.
