# Supplementary material for: Small airway dilation measured by endoscopic optical coherence tomography correlates with chronic lung allograft dysfunction
Source: J Biomed Opt. 2021 Jul 14;26(7):076005. doi: 10.1117/1.JBO.26.7.076005 (PMC8278781; doi:10.1117/1.JBO.26.7.076005)
Supplement: Supplementary file 1 [file JBO_026_076005_SD001.pdf]

SUPPLEMENTARY MATERIAL

Table S-1 Pearson correlations (R) between CLAD status and cumulative feature score for each rater. Prevalence refers to the percentage of the dataset (n=54) for each corresponding score (0,1,2), calculated per-airway. Kappas represent the overall rater reliability. Correlations that meet the significance criteria after Bonferroni correction ($p_{Bonferroni} < 0.007$) bolded.

	Feature	R (*p*-value)				Mean prevalence of scores ± SD	$\kappa_{overall}$ 95% CI
		Rater 1	Rater 2	Rater 3	Rater 4		
graduated features	DOA	0.033 (0.812)	0.094 (0.501)	0.14 (0.301)	0 (1.000)	0: 74% ± 6<br>1: 15% ± 11<br>2: 11% ± 5	**0.39 (<0.001);** 0.38 to 0.41
	ELAE	0.060 (0.664)	0.038 (0.783)	-0.072 (0.607)	0.0088 (0.950)	0: 83% ± 14<br>1: 14% ± 16<br>2: 3% ± 2	**0.26 (<0.001);** 0.25 to 0.28
	AD	**0.59 (<0.001)**	**0.40 (0.003)**	**0.49 (<0.001)**	**0.61 (<0.001)**	0: 74% ± 2<br>1: 21% ± 3<br>2: 5% ± 2	**0.48 (<0.001);** 0.47 to 0.50
binary features	AH	0.063 (0.650)	0.20 (0.147)	0.087 (0.533)	-0.021 (0.878)	0: 87% ± 7<br>1: 13% ± 7	**0.28 (<0.001);** 0.27 to 0.30
	OM	0.23 (0.090)	0.22 (0.109)	0.029 (0.838)	0.30 (0.025)	0: 87% ± 3<br>1: 13% ± 3	**0.39 (<0.001);** 0.37 to 0.41
	DLS	0.15 (0.293)	0.17 (0.220)	0.19 (0.180)	0.18 (0.180)	0: 94% ± 4<br>1: 6% ± 4	**0.33 (<0.001); 0.31 to 0.34**
